# “Broad” Impact: Perceptions of Sex/Gender-Related Psychology Journals

**DOI:** 10.3389/fpsyg.2022.796069

**Published:** 2022-03-03

**Authors:** Elizabeth R. Brown, Jessi L. Smith, Doralyn Rossmann

**Affiliations:** ^1^Department of Psychology, University of North Florida, Jacksonville, FL, United States; ^2^Department of Psychology, University of Colorado, Colorado Springs, Colorado Springs, CO, United States; ^3^Montana State University Library, Montana State University, Bozeman, MT, United States

**Keywords:** gender, androcentrism, sexism, perceptions of sex/gender research, psychological research

## Abstract

Because men are overrepresented within positions of power, men are perceived as the default in academia (androcentrism). Androcentric bias emerges whereby research by men and/or dominated by men is perceived as higher quality and gains more attention. We examined if these androcentric biases materialize within fields that study bias (psychology). How do individuals in close contact with psychology view psychology research outlets (i.e., journals) with titles including the words women, gender, sex, or feminism (sex/gender-related) or contain the words men or masculinity (men-related; Study 1) versus psychology journals that publish other-specialized research, and do these perceptions differ in the general public? While the men-related journal was less meritorious than its other-specialty journal, evidence emerged supporting androcentric bias such that the men-related journal was more favorable than the other sex/gender-related journals (Study 1). Further, undergraduate men taking psychology classes rated sex/gender-related versus other-specialty journals as less favorable, were less likely to recommend subscription (Studies 1–2), and rated the journals as lower quality (Study 2 only). Low endorsement of feminist ideology was associated with less support for sex/gender-related journals versus matched other-specialty journals (Studies 1–2). Decreased subscription recommendations for sex/gender-related journals (and the men-related journal) were mediated by decreased favorability and quality beliefs, especially for men (for the sex/gender-related journals) and those low in feminist ideology (Studies 1–2). However, we found possible androcentric-interest within the public sphere. The public reach of articles (as determined by Altmetrics) published in sex/gender-related was greater than other-specialty journals (Study 3). The consequences of these differential perceptions for students versus the public and the impact on women’s advancement in social science and psychological science are discussed.

## Introduction

In his treatise, *Truth*, Protagoras declares “of all things the measure is man,” which very explicitly centers on the experiences of men (Bonazzi, 2020). This tendency to see men and what men value as the default yields androcentric bias (Bailey et al., 2019) such as being more likely to hire and support *cis*-gendered men versus women within the academy (e.g., Moss-Racusin et al., 2012). Although the demographics of faculty at academic institutions have shifted from 66.8% men (1970) to 50% men (2018) (National Center for Education Statistics, 2020), the artifacts of androcentrism remain within academia. For instance, men, especially white straight men, are overrepresented among top academic administrators (Bichsel and McChesney, 2017; Moghimi et al., 2019), men are more likely to occupy prestigious research positions within academia (Greenbaum et al., 2018; Lobl et al., 2020; Pinho-Gomes et al., 2021), and (*cis*-white) men receive more research funding than all other groups (Witteman et al., 2019). Even within the same subfields, research associated with men is viewed as higher quality (Knobloch-Westerwick et al., 2013). Indeed, when asked to “picture a scientist” people from across generations defer to a male exemplar (Miller et al., 2018). But is this robust norm true within fields that study androcentrism, like psychology? We ask whether androcentrism extends to research *about* women, gender, and sex. The current study examines whether androcentrism is present in the evaluations and public reach of sex/gender-related research within the field of psychology.

### Androcentrism

Androcentrism is a system justifying ideology that recasts the advantages of men as a gender-neutral standard (i.e., Bem, 1993). It is the perception of men and anything related to men as default, foundational, and of focus and the perception of women and anything related to women as other and a special case (i.e., Bem, 1993). Even within an environment where women and men are equally represented, women’s gender is noticed more than men’s gender (Thomas et al., 2014), resulting in women being perceived in more gendered ways (i.e., Smith and Zarate, 1992). Because men possess higher power and status within society, we engage in categorization processes that privilege men’s experiences and values and result in androcentric bias (Bailey et al., 2019).

Androcentrism manifests in the evaluations of categories that are primarily associated with men (Bem, 1993). For instance, when job advertisements and titles contain more androcentric information, women are less likely to apply to Stout and Dasgupta (2011) and are perceived as less qualified for Hovarth and Sczesny (2016) the positions.

### Androcentrism Norms Within Research Spaces

Women within academic research face pervasive bias. For instance, women versus men science faculty are more likely to experience sexual harassment as well as gender-based discrimination, which hurts job outcomes (Settles et al., 2006). A woman as opposed to a man applying for a job as a lab manager is also less likely to be hired and is viewed as less competent (Moss-Racusin et al., 2012). Although women often research more novel topics, their research is associated with having less overall impact on the field (Hofstra et al., 2020). Thus, with few exceptions (Ceci et al., 2014), the gender bias within research output persists regardless of a country’s score on gender equity measures (Sugimoto et al., 2015). Research *by* women authors is judged as lower in quality (Knobloch-Westerwick et al., 2013) and is less likely to be: cited (Larivière et al., 2013), receive conference air time (Johnson et al., 2017), and featured in on-campus presentations (Nittrouer et al., 2018). Further, individuals from minoritized groups (e.g., white, Black, and Latinx women) are more likely to be overrepresented in research topics that have disproportionately lower citation counts (Kozlowski et al., 2022).

Also, men are more likely to be used as a baseline by which to generalize and evaluate academic tasks (see Bailey et al., 2019). This means that areas of study dominated by men (research; science; business) as compared with areas of study dominated by women (teaching; education) are more valued (Gutièrrez y Muhs et al., 2012), more prestigious (Liben et al., 2001; Watt et al., 2012), and perceived as more challenging (Liben et al., 2001; Watt et al., 2012). As such, research on other biases, as compared with research on gender bias, is more likely to be funded and appears more often in high-impact journals (e.g., Cislak et al., 2018). All told, androcentric biases emerge such that research *by* women and tasks and domains *associated with* women are marginalized. Most troubling, androcentric bias remains as a vestige even when a field becomes representationally more gender-equal because (*cis*-gendered white) men continue to be over-represented within high-ranking or highly visible roles (Klatzky et al., 2015; Vaid and Geraci, 2016).

### The Case of Psychology

Psychology has been dominated by women at the undergraduate level since the 1970s {52.7% in 1975 (National Science Foundation [NSF], 1993); 78.1% in 2012 (National Science Foundation [NSF], 2015)}. An increasing number of women with master’s degrees {42.9% in 1975 (National Science Foundation [NSF], 1993); 79.1% in 2012 (National Science Foundation [NSF], 2015)} and doctorates {31.7% in 1975 (National Science Foundation [NSF], 1993); 72.6% in 2012 (National Science Foundation [NSF], 2015)} has transformed psychology from a men-dominated field to a women-dominated field. However, notwithstanding psychological research examining androcentric bias, psychology research is deeply rooted in androcentric bias (Shields, 1975). Despite progress in gender representation, women in psychology remain underrepresented on first author publications in top journals (Brown and Goh, 2016), in awards received by divisions (Eagly and Riger, 2014; Brown and Goh, 2016), in eminence (Diener et al., 2014; Eagly and Miller, 2016), and in tenure-track positions (40.6% in 2010–2011; Oklahoma State University [OSU], 2011; see American Psychological Association Center for Workforce Studies, 2014). Further, research on the psychology of gender is often perceived by personality and/or social psychology researchers as less rigorous and mainstream than other subfields (i.e., attitudes and persuasion; judgment and decision making), and researchers who pursue research on the psychology of gender are stereotyped as being “female” (Rios and Roth, 2020).

We advance research on androcentrism by examining whether the androcentric bias materializes in the evaluation of journals that *specialize in publishing psychological research related* to women, sex, gender, and feminism (sex/gender-related). Are sex/gender-related psychology journals considered less important, impactful, and deserving of subscription recommendation than other-specialty psychology journals? Does androcentric bias look different among people within the field of psychology compared to the public writ large?

### Androcentrism and a Person’s Gender and Ideology

There is mixed evidence for whether androcentric bias changes depending on a person’s binary gender identity (Harding, 1991). *Cis*-gendered men as compared with women hold more traditional gender role attitudes (Bolzendahl and Meyers, 2004; Fodor and Balogh, 2010) and are more likely to respond negatively to or discount evidence showing gender bias (Handley et al., 2015). Due to in-group favoritism (Tajfel and Turner, 2001) and self-relevance (van Veelan et al., 2015), men compared to women are often more likely to have androcentric preferences (i.e., Bruckmüller et al., 2012; Bailey and LaFrance, 2016). On the other hand, men and women are equally likely to hold (Nosek et al., 2009) and apply implicit gender associations in discriminatory ways (Moss-Racusin et al., 2015) and often show similar levels of androcentrism (i.e., Hegarty and Buechel, 2006; Gaetano et al., 2016). We add to this literature by examining whether a person’s gender identity results in the application of androcentric biases such that research outlets related to sex/gender are perceived as less important, impactful, and deserving of a library subscription.

Research has also demonstrated individual and ideological differences in the expression of androcentrism. For example, androcentric bias is minimized to the extent that an individual is motivated to be egalitarian (Plant and Devine, 1998; Crandall et al., 2002). In contrast, when people endorse sexist ideologies, they are more likely to display androcentric bias within their language use (i.e., Swim et al., 2004; Sczesny et al., 2015). In Studies 1–2 we extend the literature on androcentrism by examining whether a different type of egalitarian belief (endorsement of feminist ideology) moderates the evaluation of sex/gender-related versus other-specialty psychology journals.

### Project Overview

Because journal impact factor implies prestige (Garfield, 2006) and quality (Saha et al., 2003), we first selected psychology research outlets related to sex/gender (and, in Study 1, a men-related journal) and other-specialty journals and matched them on impact factor. To examine whether androcentrism emerges within the field of psychology, where students and academics study androcentrism, we used a matched within-participants survey of undergraduate students enrolled in psychology classes to experimentally test how sex/gender-related (and men-related for Study 1) versus other-specialized journals faired on evaluative and behavioral expressions of bias (Studies 1–2). Next, we examined whether these androcentric biases occurred in people more distal from the field of psychology by documenting the reach of psychology research outlets through popular press metrics (Altmetrics; Study 3).

We tested several hypotheses in this series of studies. First, we tested the overall *androcentric bias hypothesis* such that the sex/gender-related psychology journals versus the matched other-specialty psychology journals (Studies 1 and 2) or men-related psychology journal (Study 1) would be perceived as less favorable, lower quality, and less recommended for subscription (Studies 1–2), and/or have less public reach (Study 3). We also examined whether the men-related journal was seen as equally or less favorable than its matched other-specialty journal. We also tested the *gender differences in androcentric bias hypothesi*s, such that men would perceive sex/gender-related journals as less meritorious than matched other-specialty psychology journals or the men-related psychology journal. Women were predicted to either favor sex/gender-related journals or show no differences in meritoriousness as compared to matched other-specialty journals or the men-related journal (Studies 1–3). We also examined whether gender differences in the perception of the men-related journal versus its matched other-specialty journal emerged. We tested the *personal ideology differences in androcentric bias hypothesis*, by examining whether participants who were lower on endorsement of feminist ideologies were especially less favorable toward sex/gender-related and men-related journals as opposed to their matched other-specialty journals (Studies 1–2). We also examined whether these same ideological differences emerged for evaluations of the men-related versus its other-specialty journal comparison. Lastly, we tested the *subscription recommendation explained by androcentric evaluative bias hypothesis* such that sex/gender-related journals versus other-specialty journals would be less likely to be recommended because they are seen as less favorable and of lower quality, especially among men and those low in feminist ideology. We also tested whether these same differences emerged for the men-related journal comparison.

## Studies 1 and 2

Because the experimental study design and the dependent variables were similar, the methods and results of Studies 1–2 are presented together.

### Method

#### Participants

Participants believed the study was spearheaded by the university library and psychology department to establish social science journal subscriptions and determine which psychology journals to prioritize. All participants were recruited from an undergraduate psychology pool in exchange for course credit (Study 1: a Mountain West University in the United States; Study 2: a Mountain West and a Southeastern University in the United States). In Study 1, one hundred ten participants (52.73% women; 84.27% white; ages 17–32, median age = 19; 10% psychology majors) were recruited, whereas in Study 2, four hundred twenty-six participants (70.10% women; 69.5% white, 8.54% Latino, 8.53% Black, 4.38% Asian, 3.38% Mixed; ages 18–60, median age = 20; 33.34% majoring in psychology [first or second major]) were recruited.

#### Journal Selection

In Study 1, we identified 31 sex/gender-related or men-related psychology journals indexed by PsycINFO with titles referring to women, sex, gender, feminism, or men. The list was filtered for psychology and/or journals that psychologists frequently publish in, based on expertise and verified by psychological researchers; we selected 4 sex/gender-related and one men-related journal. We recorded each journal’s current impact factor using Journal Citation Reports (Table 1) and identified other psychology journals indexed by PsycINFO with similar impact factors (within ± 0.011 points). When more than one journal met our criteria, the more specialized journal was selected. For instance, we matched *Feminism and Psychology* (impact factor = 0.831) with *Military Psychology* (impact factor = 0.831) versus *Social Justice Research* (impact factor = 0.829).

**TABLE 1:** Psychology journals selected for studies 1, 2, and 3 matched on impact.

Studies 1 and 3: matched impact factors as of March 2014			

Sex/gender-related journals		Other-specialty journals	

*Title*	*Impact factor*	*Title*	*Impact factor*
*Women and therapy*	0.111	*Journal of psychology in Africa*	0.109
*Feminism and psychology*	0.831	*Military psychology*	0.831
*Sex roles*	0.531	*Group processes and intergroup relations*	0.528
*Psychology of women quarterly*	0.818	*Personality and individual differences*	0.807

**Men-related journals**		**Other-specialty journal**	

***Title***	***Impact factor***	***Title***	***Impact factor***

*Psychology of men and masculinity*	0.679	*The clinical neuropsychologist*	0.678

**Study 2 and 3: matched five-year impact factors as of January 2016**			

**Sex/gender-related journals**		**Other-specialty journals**	

***Title***	***Five-year impact factor***	***Title***	***Five-year impact factor***

*Women and therapy*	0.191	*Psychologia*	0.168
*Feminism and psychology*	0.920	*Journal of classification*	0.929
*Sex roles*	2.067	*Thinking and reasoning*	2.062
*Psychology of women quarterly*	2.142	*European journal of psychological assessment*	2.124

In Study 2, we selected the same 4 sex/gender-related journals as Study 1, recorded their 5-year impact factor using Journal Citation Reports (Table 1), and determined all psychology journals in the Journal Citation Reports with similar 5-year impact factors (within ± 0.023). Given that Study 1 conflated race and class (i.e., *Journal of Psychology in Africa*), which muddles the effects as race and class are also marginalized topics of study (e.g., Kozlowski et al., 2022), in Study 2 we did not select journals that conflated race and class when more than one journal met our 5-year impact factor criteria. For instance, we chose the match for *Women and Therapy* (5-year impact factor = 0.191) to be *Psychologia* (5-year impact factor = 0.168) as opposed to the *Journal of Psychology in Africa* (5-year impact factor = 0.18).

#### Journal Type

Participants read the title and a description of each journal before completing the dependent measures. In Study 1, journal descriptions (45–324 words) were taken directly from the journal’s publication website, which replicated the naturalistic experience of participants seeking journal information (across journal comparisons word counts were within 60 words). In Study 2, journal descriptions were edited to control for word count (45–63 words).

The presentation of sex/gender-related/men-related (for Study 1) and other-specialty journals alternated using a Latin squares design. Study 1 had 10 presentation orders; Study 2 had 8 presentation orders (as there was no men-related journal comparison). Every journal had the opportunity to be reviewed first. To prevent disengagement, after rating half of the journals, participants completed a neutral break activity (word creation task and maze).

#### Androcentrism Measures: Favorability, Quality, and Subscription Recommendations

Participants rated their favorability toward the journals on six items (modified from Handley et al., 2015; i.e., “To what extent is this journal important to have in our [university initials] library”; Table 2) on scales ranging from 1 (*not at all*) to 6 (*very much*). Ratings were averaged for each journal (α’s ≥ 0.86; Table 2).

**TABLE 2:** Favorability items and Cronbach’s alphas for studies 1 and 2.

Favorability Items (Handley et al., 2015)									
To what extent is this journal important to have in our [university initials] library?									
To what extent would you expect that the research in this journal would be of high quality?									
To what extent would you expect the articles in this journal to make significant contributions to advancing the field of psychology?									
To what extent do the contents of this journal sound interesting?									
To what extent would reading research published in this journal be useful to you?									
Overall, my evaluation of this journal is favorable.									

**Cronbach’s alphas for studies 1 and 2**									

	***Women and therapy***		***Feminism and psychology***		***Sex roles***		***Psychology of women quarterly***		***Psychology of men and masculinity***
					
	**Study 1**	**Study 2**	**Study 1**	**Study 2**	**Study 1**	**Study 2**	**Study 1**	**Study 2**	**Study 1**

Favorability	0.89	0.93	0.90	0.93	0.91	0.93	0.92	0.93	0.89
Quality	0.98	–	0.96	–	0.97	–	0.98	–	0.91

	**Matched other-specialty**		**Matched other-specialty**		**Matched other-specialty**		**Matched other-specialty**		**Matched other-specialty**

Favorability	0.89	0.90	0.87	0.87	0.88	0.89	0.91	0.90	0.86
Quality	0.98	–	0.98	–	0.97	–	0.99	–	0.91

**Feminist ideology items** (Fisher et al., 2000; original items taken from Bargad and Hyde (1991), Reid and Purcell (2004); study 1: α = 0.94; study 2: α=0.93).									

I am very committed to a cause that I believe contributes to a more fair and just world for all people.									
I want to work to improve women’s status.									
I am willing to make certain sacrifices to effect change in this society in order to create a nonsexist, peaceful place where all people have equal opportunities.									
It is very satisfying to me to be able to use my talents and skills in my work in the women’s movement.									
I care very deeply about men and women having equal opportunities in all respects.									
I choose my “causes” carefully to work for great equality for all people.									
I feel that I am very powerful and effective spokesperson for the women’s issues I am concerned with right now.									
One some level, my motivation for almost every activity I engage in is my desire of an egalitarian world.									
I owe it not only to women but to all people who work for greater opportunity and equality for all.									
I am a feminist.									
Being a feminist is central to who I am.									
I would be proud to be identified as a feminist.									

Participants ranked the quality of the journals (“I would rank this journal in the _____ percentile on quality”) by choosing a number ranging from the 5th (l*owest*) to the 99th (*highest*) quality. In Study 1, participants also ranked how other students would rank the quality of the journals (“I predict _____ that other students at [university initials] would likely rank this journal in the _____ percentile on quality”) by choosing a number ranging from the 5th (l*owest*) to the 99th (*highest*) quality. Ratings for Study 1 were averaged for each journal (α’s ≥ 0.91; Table 2).

Participants also made journal subscription recommendations. In Study 1, participants took “everything into consideration” and determined the percentile ranking of the likelihood [university initials] library should maintain this journal subscription from 0% (*no chance*) to 100% (*definitely*). In Study 2, participants rated the likelihood that the [university initials] library would maintain this journal subscription from 0% (*no chance*) to 100% (*definitely*) relative to all journals in psychology.

#### Ideological Measure: Feminist Ideology

Participants completed nine feminist identity items on scales ranging from 1 (*strongly disagree*) to 7 (*strongly agree*; Fisher et al., 2000; original items taken from Bargad and Hyde, 1991). Items included: “I am very committed to a cause that I believe contributes to a more fair and just world for all people” (Table 2). Participants also completed 3 items on scales ranging from 1 (*strongly disagree*) to 7 (*strongly agree*) about whether they self-identified as a feminist taken from the Social Identity subscale of the Social Identity Specific Collectivism scale (Reid and Purcell, 2004). Items included “I am a feminist” (Table 2). All 12 items were averaged to create a feminist ideology composite (α’s ≥ 0.93; Table 2).

### Results

Given that there were four sex/gender-related journals and 1 men-related journal, the sex/gender-related journals could not be submitted to the same mixed analysis of variance (ANOVA) as the men-related journal. Thus, participants’ evaluations of journal comparisons for sex/gender-related (versus matched other-specialty) were separately examined through 2 (journal type: sex/gender-related versus matched other-specialty) × 4 (matched comparisons at the level of the journal) × 2 (participant gender) mixed ANOVAs with journal type and matched comparisons at the level of the journal as within-participants variables. Participants’ evaluations of the journal comparisons for the men-related journal (versus its matched other-specialty journal) were examined through 2 (journal type: men-related versus matched other-specialty) × 2 (participant gender) mixed ANOVAs with journal type as a within-participants variable. Comparisons for sex/gender-related journals (combined) versus the men-related journal were separately examined through 2 [journal type: sex/gender-related journals (combined) versus men-related journal] × 2 (participant gender) mixed ANOVAs with journal type as a within-participants variable. For both Studies 1 and 2, we completed *post hoc* power analyses using G*Power to determine whether the analyzed samples had sufficient power to detect the main effects of journal and the moderation by gender.

To examine whether feminist ideology moderated our effects, first we examined whether gender differences emerged in participants’ ratings of feminist ideology, then we examined the relationship between the ratings of each journal type and feminist ideology, and finally we used Fisher’s *r*-to-*z* test to examine the differences between the correlation coefficients.

We employed regressions to examine whether the differential journal subscription recommendations were mediated by decreased favorability and quality beliefs toward sex/gender-related journals and the men-related journal.

Across both studies, we only report the main effects of journal type and interactions between journal type and gender. Other main effects, interactions, means, and standard deviations are detailed in Tables 3–6. In Studies 1–3, effect sizes with positive numbers indicate differences favoring other-specialty journals and the men-related journal.

**TABLE 3:** ANOVAs comparing sex/gender-related to other matched specialty journals: study 1.

	Favorability (*df*: 3, 106)					Quality (*df*: 3, 104)					Subscription recommendations (*df*: 3, 104)				
			
	*F*	*p*	*d*	*M*	*SD*	*F*	*p*	*d*	*M*	*SD*	*F*	*p*	*d*	*M*	*SD*
Gender	3.37	0.069	−0.35	–	–	0.52	0.472	0.19	–	–	0.40	0.531	−0.14	–	–
Men	–	–	–	3.85	0.73	–	–	–	56.97	21.89	–	–	–	50.40	18.18
Women	–	–	–	4.09	0.64	–	–	–	52.50	25.01	–	–	–	52.86	17.49
Journal	0.26	0.612	0.03	–	–	0.00	0.958	−0.001	–	–	0.32	0.574	0.03	–	–
Sex/gender-related	–	–	–	3.96	1.12	–	–	–	55.29	29.55	–	–	–	51.33	30.20
Matched other-specialty	–	–	–	3.99	1.00	–	–	–	55.26	29.21	–	–	–	52.16	29.43
Type	6.65	<0.001	0.06–0.22	–	–	2.88	0.036	0.003–0.17	–	–	6.28	<0.001	0.01–0.27	–	–
*Women and Therapy* Comparisons	–	–	–	3.92	1.02	–	–	–	55.92	29.43	–	–	–	48.91	30.25
*Feminism and Psychology* Comparisons	–	–	–	3.98	1.04	–	–	–	57.14	29.16	–	–	–	52.97	28.30
*Sex Roles* Comparisons	–	–	–	3.85	1.05	–	–	–	52.22	28.21	–	–	–	48.51	29.43
*Psychology of Women Quarterly* Comparisons	–	–	–	4.15	1.10	–	–	–	55.83	30.58	–	–	–	56.60	30.65
Gender × Journal	25.01	<0.001	–	–	–	3.84	0.053	–	–	–	8.43	0.005	–	–	–
Men’s evaluations	12.78	<0.001	0.37	–	–	1.63	0.208	0.11	–	–	4.88	0.032	0.23	–	–
Sex/gender-related	–	–	–	3.65	1.13	–	–	–	55.44	29.44	–	–	–	46.99	30.64
Matched other-specialty	–	–	–	4.05	1.02	–	–	–	58.50	28.55	–	–	–	54.16	31.06
Women’s evaluations	12.11	0.001	−0.33	–	–	2.32	0.134	−0.10	–	–	3.40	0.070	−0.17	–	–
Sex/gender-related	–	–	–	4.26	1.03	–	–	–	55.16	29.71	–	–	–	55.21	29.32
Matched other-specialty	–	–	–	3.93	0.97	–	–	–	52.26	29.57	–	–	–	50.38	27.84
Gender × Type	2.36	0.071	–	–	–	2.80	0.040	–	–	–	1.68	0.172	–	–	–
Men’s evaluations	6.93	<0.001	0.10–0.36	–	–	4.90	0.003	0.01–0.36	–	–	4.90	0.003	0.13–0.38	–	–
*Women and Therapy* Comparisons	–	–	–	3.76	1.08	–	–	–	57.87	29.56	–	–	–	49.02	31.77
*Feminism and Psychology* Comparisons	–	–	–	3.96	1.08	–	–	–	60.99	28.05	–	–	–	52.99	29.58
*Sex Roles* Comparisons	–	–	–	3.65	1.11	–	–	–	50.75	28.28	–	–	–	44.20	30.85
*Psychology of Women Quarterly* Comparisons	–	–	–	4.04	1.08	–	–	–	58.27	29.62	–	–	–	56.10	31.02
Women’s evaluations	2.44	0.066	0.04–0.23	–	–	0.02	1.00	0.00–0.02	–	–	2.72	0.046	0.02–0.28	–	–
*Women and Therapy* Comparisons	–	–	–	4.08	0.95	–	–	–	54.10	29.42	–	–	–	48.82	28.96
*Feminism and Psychology* Comparisons	–	–	–	4.00	1.01	–	–	–	53.58	29.85	–	–	–	52.96	27.23
*Sex Roles* Comparisons	–	–	–	4.04	0.97	–	–	–	53.59	28.21	–	–	–	52.36	27.68
*Psychology of Women Quarterly* Comparisons	–	–	–	4.25	1.12	–	–	–	53.57	31.41	–	–	–	57.04	30.45
Journal × Type	23.93	<0.001	–	–	–	14.12	<0.001	–	–	–	28.65	<0.001	–	–	–
Sex/gender-related	12.00	<0.001	0.09–0.49	–	–	7.12	<0.001	0.05–0.32	–	–	11.27	<0.001	0.15–0.56	–	–
*Women and Therapy* Comparisons	–	–	–	4.18	1.02	–	–	–	61.44	29.46	–	–	–	58.28	29.86
*Feminism and Psychology* Comparisons	–	–	–	3.67	1.08	–	–	–	51.99	28.91	–	–	–	42.31	27.19
*Sex Roles* Comparisons	–	–	–	3.95	1.14	–	–	–	53.12	28.71	–	–	–	50.09	30.40
*Psychology of Women Quarterly* Comparisons	–	–	–	4.06	1.18	–	–	–	54.61	30.58	–	–	–	54.66	31.23
Matched other-specialty	17.98	<0.001	0.04–0.65	–	–	9.03	<0.001	0.03–0.42	–	–	25.58	<0.001	0.18–0.91	–	–
*Women and Therapy* Comparisons	–	–	–	3.67	0.97	–	–	–	50.39	28.48	–	–	–	39.55	27.75
*Feminism and Psychology* Comparisons	–	–	–	4.28	0.90	–	–	–	62.29	28.64	–	–	–	63.63	25.31
*Sex Roles* Comparisons	–	–	–	3.75	0.95	–	–	–	51.32	27.81	–	–	–	46.92	28.49
*Psychology of Women Quarterly* Comparisons	–	–	–	4.24	1.02	–	–	–	57.05	30.67	–	–	–	58.54	30.08
Gender × Journal × Type	2.75	0.043	–	–	–	1.52	0.208	–	–	–	2.19	0.089	–	–	–
Men’s evaluations	16.47	<0.001	–	–	–	11.23	<0.001	–	–	–	18.08	<0.001	–	–	–
Sex/gender-related	4.97	0.003	0.06–0.41	–	–	5.15	0.002	0.005–0.47	–	–	7.78	<0.001	0.06–0.65	–	–
*Women and Therapy* Comparisons	–	–	–	3.92	1.12	–	–	–	63.89	29.07	–	–	–	58.56	31.36
*Feminism and Psychology* Comparisons	–	–	–	3.47	1.10	–	–	–	53.75	28.89	–	–	–	39.41	27.75
*Sex Roles* Comparisons	–	–	–	3.54	1.19	–	–	–	50.20	29.67	–	–	–	41.12	30.79
*Psychology of Women Quarterly* Comparisons	–	–	–	3.65	1.07	–	–	–	53.90	29.20	–	–	–	48.88	29.62
Matched other-specialty	16.75	<0.001	0.01–0.92	–	–	10.01	<0.001	0.02–0.64	–	–	16.64	<0.001	0.12–0.99	–	–
*Women and Therapy* Comparisons	–	–	–	3.59	1.02	–	–	–	51.85	28.87	–	–	–	39.48	29.51
*Feminism and Psychology Comparisons*	–	–	–	4.44	0.81	–	–	–	68.22	25.45	–	–	–	66.56	24.93
*Sex Roles* Comparisons	–	–	–	3.75	1.02	–	–	–	51.30	27.11	–	–	–	47.28	30.92
*Psychology of Women Quarterly* Comparisons	–	–	–	4.43	0.96	–	–	–	62.64	29.68	–	–	–	63.32	30.98
Women’s evaluations	9.77	<0.001	–	–	–	4.26	0.006	–	–	–	12.27	<0.001	–	–	–
Sex/gender-related	9.56	<0.001	0.02–0.53	–	–	3.25	0.023	0.02–0.30	–	–	5.90	<0.001	0.25–0.82	–	–
*Women and Therapy* Comparisons	–	–	–	4.41	0.86	–	–	–	59.17	29.90	–	–	–	39.61	26.35
*Feminism and Psychology* Comparisons	–	–	–	3.85	1.04	–	–	–	50.36	29.09	–	–	–	61.02	25.58
*Sex Roles* Comparisons	–	–	–	4.33	0.95	–	–	–	55.84	27.78	–	–	–	46.61	26.42
*Psychology of Women Quarterly * Comparisons	–	–	–	4.43	1.16	–	–	–	55.27	32.07	–	–	–	54.27	28.86
Matched other-specialty	3.71	0.013	0–0.42	–	–	1.50	0.216	0.02–0.26	–	–	9.37	<0.001	0.003–0.51	–	–
*Women and Therapy* Comparisons	–	–	–	3.75	0.92	–	–	–	49.04	28.30	–	–	–	58.03	28.74
*Feminism and Psychology* Comparisons	–	–	–	4.14	0.96	–	–	–	56.79	30.50	–	–	–	44.89	26.65
*Sex Roles* Comparisons	–	–	–	3.75	0.90	–	–	–	51.34	28.71	–	–	–	58.11	27.94
*Psychology of Women Quarterly* Comparisons	–	–	–	4.07	1.06	–	–	–	51.86	30.93	–	–	–	59.81	31.98

**TABLE 4:** ANOVAs comparing the men-related to the matched other-specialty journal: study 1.

	Favorability (*df*: 1, 107)					Quality (*df*: 1, 105)					Subscription recommendations (*df*: 1, 107)				
			
	*F*	*p*	*d*	*M*	*SD*	*F*	*p*	*d*	*M*	*SD*	*F*	*p*	*d*	*M*	*SD*
Gender	0.64	0.426	−0.15	–	–	0.86	0.355	0.18	–	–	0.17	0.685	−0.08	–	–
Men	–	–	–	4.36	0.80	–	–	–	65.39	24.26	–	–	–	60.19	23.77
Women	–	–	–	4.47	0.68	–	–	–	60.68	27.78	–	–	–	61.82	18.03
Journal	19.92	<0.001	0.52	–	–	31.08	<0.001	0.42	–	–	13.68	<0.001	0.47	–	–
Men-related	–	–	–	4.18	0.99	–	–	–	56.96	28.03	–	–	–	54.52	28.62
Matched other-specialty	–	–	–	4.66	0.86	–	–	–	68.89	28.68	–	–	–	67.57	26.76
Gender × Journal	0.36	0.551	–	–	–	0.03	0.859	–	–	–	0.33	0.564	–	–	–
Men’s evaluations	7.86	0.007	0.44	–	–	13.75	<0.001	0.43	–	–	4.54	0.038	0.36	–	–
Men-related	–	–	–	4.15	1.03	–	–	–	59.62	27.20	–	–	–	54.72	31.43
Matched other-specialty	–	–	–	4.57	0.89	–	–	–	71.16	26.14	–	–	–	65.65	28.75
Women’s evaluations	12.40	<0.001	0.61	–	–	17.54	<0.001	0.41	–	–	9.81	0.003	0.59	–	–
Men-related	–	–	–	4.20	0.96	–	–	–	54.53	28.80	–	–	–	54.33	26.08
Matched other-specialty	–	–	–	4.75	0.83	–	–	–	66.83	30.91	–	–	–	69.32	24.94

**TABLE 5:** ANOVAs comparing men-related to the sex/gender-related journals: study 1.

	Favorability (*df*: 1, 107)					Quality (*df*: 1, 105)					Subscription recommendations (*df*: 1, 107)				
			
	*F*	*p*	*d*	*M*	*SD*	*F*	*p*	*d*	*M*	*SD*	*F*	*p*	*d*	*M*	*SD*
Gender	3.78	0.055	−0.36	–	–	0.41	0.525	0.12	–	–	0.84	0.363	−0.16	–	–
Men	–	–	–	3.91	0.92	–	–	–	57.53	23.89	–	–	–	50.99	22.59
Women	–	–	–	4.22	0.79	–	–	–	54.48	26.65	–	–	–	54.47	20.48
Journal	8.02	0.006	0.22	–	–	1.69	0.197	0.08	–	–	1.57	0.213	0.12	–	–
Men-related	–	–	–	4.18	0.99	–	–	–	56.96	28.03	–	–	–	54.52	28.62
Sex/gender-related	–	–	–	3.97	0.94	–	–	–	54.80	25.83	–	–	–	51.42	22.07
Gender × Journal	12.50	<0.001	–	–	–	1.25	0.266	–	–	–	2.51	0.116	–	–	–
Men’s evaluations	18.70	<0.001	0.49	–	–	2.30	0.136	0.16	–	–	3.29	0.075	0.28	–	–
Men-related	–	–	–	4.15	1.03	–	–	–	59.62	27.20	–	–	–	54.72	31.43
Sex/gender-related	–	–	–	3.66	0.97	–	–	–	55.44	24.40	–	–	–	47.27	21.69
Women’s evaluations	0.27	0.606	−0.06	–	–	0.02	0.885	0.01	–	–	0.07	0.795	−0.04	–	–
Men-related	–	–	–	4.20	0.96	–	–	–	54.53	28.80	–	–	–	54.33	26.08
Sex/gender-related	–	–	–	4.25	0.81	–	–	–	54.22	27.28	–	–	–	55.21	21.91

**TABLE 6:** ANOVAs comparing sex/gender-related to other matched specialty journals: study 2.

	Favorability (*df*: 3, 411)					Quality (*df*: 3, 406)					Subscription recommendations (*df*: 3, 407)				
			
	*F*	*p*	*d*	*M*	*SD*	*F*	*p*	*d*	*M*	*SD*	*F*	*p*	*d*	*M*	*SD*
Gender	8.83	0.003	−0.33	–	–	2.19	0.140	0.16	–	–	0.94	0.333	−0.11	–	–
Men	–	–	–	3.80	0.65	–	–	–	62.31	17.97	–	–	–	63.02	15.43
Women	–	–	–	4.03	0.73	–	–	–	59.00	23.49	–	–	–	64.76	15.67
Journal	19.93	<0.001	0.06	–	–	23.18	<0.001	0.08	–	–	21.42	<0.001	0.12	–	–
Sex/gender-related	–	–	–	3.92	1.19	–	–	–	58.42	30.20	–	–	–	62.34	26.47
Matched other-specialty	–	–	–	3.99	1.14	–	–	–	60.97	30.82	–	–	–	65.51	27.28
Type	134.49	<0.001	0.09–0.78	–	–	56.01	<0.001	0.05–0.49	–	–	68.67	<0.001	0.12–0.63	–	–
*Women and Therapy* Comparisons	–	–	–	3.99	1.16	–	–	–	60.38	30.33	–	–	–	64.81	26.83
*Feminism and Psychology* Comparisons	–	–	–	3.53	1.14	–	–	–	52.57	30.39	–	–	–	56.17	27.55
*Sex Roles* Comparisons	–	–	–	4.41	1.07	–	–	–	67.07	29.25	–	–	–	73.02	26.27
*Psychology of Women Quarterly* Comparisons	–	–	–	3.88	1.13	–	–	–	58.78	30.43	–	–	–	61.71	27.05
Gender × Journal	58.82	<0.001	–	–	–	39.45	<0.001	–	–	–	20.97	<0.001	–	–	–
Men’s evaluations	55.15	<0.001	0.57	–	–	39.49	<0.001	0.49	–	–	26.26	<0.001	0.40	–	–
Sex/gender-related	–	–	–	3.50	1.12	–	–	–	56.07	28.40	–	–	–	57.63	27.08
Matched other-specialty	–	–	–	4.09	0.95	–	–	–	69.25	25.16	–	–	–	67.98	24.27
Women’s evaluations	8.39	0.004	−0.13	–	–	1.88	0.172	−0.05	–	–	0.00	0.963	0.002	–	–
Sex/gender-related	–	–	–	4.10	1.18	–	–	–	59.44	30.90	–	–	–	64.39	25.94
Matched other-specialty	–	–	–	3.94	1.22	–	–	–	57.83	32.47	–	–	–	64.44	28.43
Gender × Type	3.13	0.025	–	–	–	0.67	0.573	–	–	–	5.82	<0.001	–	–	–
Men’s evaluations	47.59	<0.001	0.04–0.71	–	–	18.41	<0.001	0.03–0.47	–	–	17.29	<0.001	0.05–0.46	–	–
*Women and Therapy* Comparisons	–	–	–	3.78	1.09	–	–	–	62.21	26.98	–	–	–	61.79	26.89
*Feminism and Psychology* Comparisons	–	–	–	3.47	1.01	–	–	–	56.25	28.13	–	–	–	58.82	25.88
*Sex Roles* Comparisons	–	–	–	4.20	1.06	–	–	–	68.90	25.73	–	–	–	70.28	23.35
*Psychology of Women Quarterly* Comparisons	–	–	–	3.74	1.02	–	–	–	61.26	27.78	–	–	–	60.31	27.20
Women’s evaluations	131.12	<0.001	0.13–0.85	–	–	53.43	<0.001	0.06–0.49	–	–	83.45	<0.001	0.14–0.74	–	–
*Women and Therapy* Comparisons	–	–	–	4.09	1.17	–	–	–	59.59	31.66	–	–	–	66.12	26.73
*Feminism and Psychology* Comparisons	–	–	–	3.55	1.20	–	–	–	50.98	31.20	–	–	–	55.02	28.19
*Sex Roles* Comparisons	–	–	–	4.51	1.06	–	–	–	66.28	30.64	–	–	–	74.21	23.15
*Psychology of Women Quarterly* Comparisons	–	–	–	3.94	1.17	–	–	–	57.70	31.47	–	–	–	62.32	26.99
Journal × Type	44.60	<0.001	–	–	–	19.07	<0.001	–	–	–	32.32	<0.001	–	–	–
Sex/gender-related	17.37	<0.001	0.05–0.29	–	–	9.25	<0.001	0.03–0.21	–	–	10.39	<0.001	0.04–0.27	–	–
*Women and Therapy Comparisons*	–	–	–	3.85	1.23	–	–	–	56.79	30.41	–	–	–	59.80	27.57
*Feminism and Psychology* Comparisons	–	–	–	3.79	1.19	–	–	–	56.02	30.34	–	–	–	60.79	26.92
*Sex Roles* Comparisons	–	–	–	4.13	1.14	–	–	–	62.22	29.81	–	–	–	66.94	24.42
*Psychology of Women Quarterly* Comparisons	–	–	–	3.91	1.18	–	–	–	58.66	29.96	–	–	–	61.82	26.39
Matched other-specialty	154.45	0<.001	0.56–1.47	–	–	60.55	<0.001	0.17–0.44	–	–	87.39	<0.001	0.31–1.14	–	–
*Women and Therapy* Comparisons	–	–	–	4.14	1.06	–	–	–	63.97	29.86	–	–	–	69.82	25.13
*Feminism and Psychology* Comparisons	–	–	–	3.27	1.03	–	–	–	49.11	30.07	–	–	–	51.54	27.44
*Sex Roles* Comparisons	–	–	–	4.69	0.90	–	–	–	71.92	27.89	–	–	–	79.10	20.34
*Psychology of Women Quarterly* Comparisons	–	–	–	3.86	1.08	–	–	–	58.89	30.93	–	–	–	61.60	27.73
Gender × Journal × Type	8.47	<0.001	–	–	–	3.48	<0.001	–	–	–	5.98	<0.001	–	–	–
Men’s evaluations	9.42	<0.001	–	–	–	3.39	0.018	–	–	–	6.91	<0.001	–	–	–
Sex/gender-related	12.01	<0.001	0–0.44	–	–	4.87	0.003	0.08–0.31	–	–	5.06	0.002	0.07–0.35	–	–
*Women and Therapy* Comparisons	–	–	–	3.33	1.07	–	–	–	54.33	27.90	–	–	–	53.48	27.52
*Feminism and Psychology* Comparisons	–	–	–	3.33	1.07	–	–	–	52.07	28.60	–	–	–	56.11	27.04
*Sex Roles* Comparisons	–	–	–	3.82	1.15	–	–	–	60.85	28.20	–	–	–	62.79	25.11
*Psychology of Women Quarterly* Comparisons	–	–	–	3.52	1.12	–	–	–	57.02	28.49	–	–	–	58.12	28.03
Matched other-specialty	45.38	<0.001	0.29–1.12	–	–	16.96	<0.001	0.18–0.69	–	–	19.92	<0.001	0.04–0.75	–	–
*Women and Therapy* Comparisons	–	–	–	4.23	0.91	–	–	–	70.10	23.63	–	–	–	70.10	23.56
*Feminism and Psychology* Comparisons	–	–	–	3.60	0.93	–	–	–	60.43	27.12	–	–	–	61.52	24.49
Sex roles comparisons	–	–	–	4.58	0.81	–	–	–	76.95	20.09	–	–	–	77.77	18.75
*Psychology of Women Quarterly* Comparisons	–	–	–	3.97	0.86	–	–	–	65.51	26.49	–	–	–	62.51	26.27
Women’s evaluations	65.44	<0.001	–	–	–	29.06	<0.001	–	–	–	46.80	<0.001	–	–	–
Sex/gender-related	6.86	<0.001	0.008–0.24	–	–	4.79	0.003	0.004–0.17	–	–	6.60	<0.001	0.01–0.24	–	–
*Women and Therapy* Comparisons	–	–	–	4.08	1.22	–	–	–	57.86	31.42	–	–	–	62.55	27.18
*Feminism and Psychology* Comparisons	–	–	–	3.99	1.19	–	–	–	57.73	30.95	–	–	–	62.83	26.65
*Sex Roles* Comparisons	–	–	–	4.27	1.12	–	–	–	62.81	30.51	–	–	–	68.75	23.93
*Psychology of Women Quarterly* Comparisons	–	–	–	4.07	1.17	–	–	–	59.38	30.60	–	–	–	63.42	25.53
Matched other-specialty	174.09	<0.001	0.25–1.64	–	–	68.31	<0.001	0.17–0.84	–	–	115.02	<0.001	0.31–1.33	–	–
*Women and Therapy* Comparisons	–	–	–	4.10	1.12	–	–	–	61.33	31.86	–	–	–	69.69	25.83
*Feminism and Psychology* Comparisons	–	–	–	3.12	1.03	–	–	–	44.23	30.01	–	–	–	47.20	27.55
*Sex Roles* Comparisons	–	–	–	4.74	0.94	–	–	–	69.74	30.42	–	–	–	79.67	21.00
*Psychology of Women Quarterly* Comparisons	–	–	–	3.82	1.16	–	–	–	56.03	32.29	–	–	–	61.21	28.38

*Note for **Tables 3–6**. The convention of Cohen’s d was used: <0.20 a small effect, 0.20 to 0.80 a moderate effect, >0.80 a large effect. Effect sizes with positive numbers indicate differences favoring other-specialty journals and men.*

#### Favorability and Subscription Recommendations

##### Testing the Androcentric Bias Hypothesis and *post hoc* Power Analyses

Sex/gender-related and the men-related journal [versus their matched other-specialty journal(s)] were perceived less favorably [Sex/gender-related, Study 2: *F*(1,411) = 19.93, *p* < 0.001, *d* = 0.06; Men-related, Study 1: *F*(1,107) = 19.92, *p* < 0.001, *d* = 0.52], as lower quality [Sex/gender-related, Study 2: *F*(1,406) = 23.18, *p* < 0.001, *d* = 0.08; Men-related, Study 1: *F*(1,105) = 31.08, *p* < 0.001, *d* = 0.42], and having lower subscription recommendations [Sex/gender-related, Study 2: *F*(1,407) = 21.42, *p* < 0.001, *d* = 0.12; Men-related, Study 1: *F*(1,107) = 13.68, *p* < 0.001, *d* = 0.47]. No significant effects of journal type emerged between sex/gender-related versus other-specialty journals in Study 1 [favorability: *F*(1,106) = 0.26, *p* = 0.612, *d* = 0.03; quality: *F*(1,104) = 0.00, *p* = 0.958, *d* = –0.001; subscription recommendations: *F*(1,104) = 0.32, *p* = 0.574, *d* = 0.03]. Although the men-related journal was perceived more favorably than the sex/gender-related journals [*F*(1,107) = 8.02, *p* = 0.006, *d* = 0.22], no differences emerged when comparing the men-related journal to the sex/gender-related journals for quality [*F*(1,105) = 1.69, *p* = 0.197, *d* = 0.08] and subscription recommendations [*F*(1,107) = 2.51, *p* = 0.116, *d* = 0.12].

While the analyzed sample in Study 2 had sufficient power to detect the main effect of journal for sex/gender-related journal comparisons and the analyzed sample in Study 1 had sufficient power to detect the main effect of journal for the men-related journal comparisons, the analyzed sample in Study 1 did not have sufficient power to detect the main effect of journal for the sex/gender-related journal comparisons (see Table 7).

**TABLE 7:** *Post hoc* power analyses for the main effects and interactions for studies 1 and 2.

	Study 1			Study 2		
	Partial *η*^2^	Repeated measures *rs*	Power	Partial *η*^2^	Repeated measures *rs*	Power
Main effect of Journal						
Sex/gender-related						
Favorability	0.002	0.147–0.294	0.140–0.163	0.046	0.121–0.346	1.00
Quality	0.000	0.567–0.616	0.052–0.052	0.054	0.369–0.369	1.00
Subscription Recommendations	0.003	0.117–0.312	0.171–0.241	0.050	0.149–0.284	1.00
Men-related						
Favorability	0.157	0.263	1.00	–	–	–
Quality	0.228	0.700	1.00	–	–	–
Subscription Recommendations	0.113	0.136	1.00	–	–	–
Journal Type × Participant Gender						
Men						
Sex/gender-related						
Favorability	0.200	0.158–0.439	1.00	0.310	0.003–0.334	1.00
Quality		0.431–0.605	0.96–1.00	0.245	0.266–0.447	1.00
Subscription Recommendations		0.015–0.403	1.00	0.176	0.093–0.248	1.00
Men-related						
Favorability	0.134	0.376	1.00	–	–	–
Quality	0.216	0.654	1.00	–	–	–
Subscription Recommendations	0.082	0.247	1.00	–	–	–
Women						
Sex/gender-related						
Favorability	0.180	0.101–0.487	1.00	0.028	0.222–0.278	1.00
Quality	0.041	0.566–0.714	1.00	0.007	0.440–0.606	0.99–1.00
Subscription Recommendations	0.058	0.191–0.361	1.00	0.000	0.124–0.308	0.08–0.09
Men-related						
Favorability	0.181	0.142	1.00	–	–	–
Quality	0.242	0.731	1.00	–	–	–
Subscription Recommendations	0.149	−0.001	1.00	–	–	–

##### Testing the Gender Differences in Androcentric Bias Hypothesis and *post hoc* Power Analyses

**FIGURE 1:**
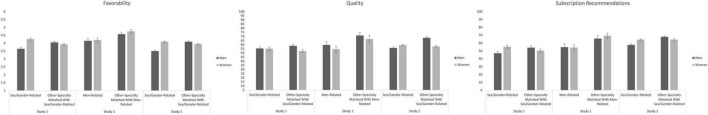
Students reactions toward sex/gender-related versus other-specialty and men-related versus other-specialty psychology publications (Studies 1 and 2). Error bars represent standard errors. Favorability was rated on scales ranging from 1 (*not at all*) to 6 (*very much*); quality was rated on scales ranging from 5th (*lowest*) to 99th (*highest*); subscription maintenance recommendations were made on scales ranging from 0% (*no chance*) to 100% (*definitely*).

When examining the sex/gender-related journals versus the other-specialty psychology journals, significant Journal Type × Participant Gender interactions emerged for favorability [Study 1: *F*(1,106) = 25.01, *p* < 0.001; Study 2: *F*(1,411) = 58.82, *p* < 0.001], quality [Study 2: *F*(1,406) = 23.18, *p* < 0.001], and subscription recommendations [Study 1: *F*(1,104) = 8.43, *p* = 0.005; Study 2: *F*(1,407) = 20.97, *p* < 0.001; Figure 1]. Men viewed sex/gender-related versus other-specialty journals less favorably (Study 1: *p* < 0.001, *d* = 0.37; Study 2: *p* < 0.001, *d* = 0.57), of lower quality (Study 2: *p* < 0.001, *d* = 0.49), and were less likely to be recommended for subscription (Study 1: *p* = 0.032, *d* = 0.23; Study 2: *p* < 0.001, *d* = 0.40). Although women viewed sex/gender-related versus other-specialty journals more favorably (Study 1: *p* = 0.001, *d* = –0.33; Study 2: *p* = 0.004, *d* = –0.13), no differences between sex/gender-related and other-specialty journals emerged for quality (Study 2: *p* = 0.172, *d* = –0.05) and subscription recommendations (Study 1: *p* = 0.070, *d* = –0.17; Study 2: *p* = 0.963, *d* = 0.002) (Figure 1).

No Journal Type × Participant Gender interaction emerged when examining the sex/gender-related journals versus the other-specialty journals for quality [Study 1, *F*(1,104) = 3.84, *p* = 0.053] or when examining the men-related journal versus its matched other-specialty journal [favorability: *F*(1,107) = 0.36, *p* = 0.551; quality: *F*(1,105) = 0.03, *p* = 0.859; subscription recommendations: *F*(1,107) = 0.33, *p* = 0.564].

However, when comparing the men-related journal to the sex/gender-related journals, a Participant Gender × Journal Type interaction emerged for favorability [*F*(1,107) = 12.50, *p* < 0.001] but not quality [*F*(1,105) = 1.25, *p* = 0.266] or subscription recommendations [*F*(1,107) = 2.51, *p* = 0.116]. Men were less favorable (*p* < 0.001, *d* = 0.49) but women were equally favorable (*p* = 0.606, *d* = –0.06) when comparing the sex/gender-related journals to the men-related journal.

Most importantly, the analyzed samples in both Studies 1 and 2 had the power to detect simple main effects of the journal comparisons (sex/gender-related and men-related) for men and women participants with the exception of women’s subscription recommendations in Study 2 (see Table 7).

##### Testing the Personal Ideology Differences in Androcentric Bias Hypothesis

Although women (Study 1: *M* = 4.57, *SD* = 1.30; Study 2: *M* = 4.82, *SD* = 1.20) were more likely to endorse a feminist ideology than men (Study 1: *M* = 4.03, *SD* = 1.12; Study 2: *M* = 4.13, *SD* = 1.13) [Study 1: *F*(1,101) = 5.07, *p* = 0.027, *d* = –0.45 (95% CI 3.66–4.04); Study 2: *F*(1,416) = 30.01, *p* < 0.001, *d* = –0.59 (95% CI 0.444 –0.940)], we examined whether participants who were lower on endorsement of feminist ideologies were especially less favorable toward sex/gender-related and men-related journals as opposed to their matched other-specialty journals. In both Studies 1 and 2, the correlation between feminist ideology and the journal type was weaker for other-specialty versus sex/gender-related journals (see Table 8). For the men-related journal, while the correlation between feminist ideology and journal type was weaker for other-specialty versus men-related journals for favorability, no differences between correlations emerged for quality and subscription recommendations (see Table 8).

**TABLE 8:** Examining feminist ideology effects: correlations between feminist ideology and journal type.

	Study 1			Study 2		
	
	Sex/gender-related Fisher’s *z*	Matched other-specialty Fisher’s *z*	*p*	Sex/gender-related Fisher’s *z*	Matched other-specialty Fisher’s *z*	*p*
**Sex/gender-related journal comparisons**						
Favorability	0.804	0.257	0.001	0.652	0.166	<0.001
Quality	0.349	0.094	0.003	0.430	0.085	<0.001
Subscription Recommendations	0.419	−0.010	<0.001	0.341	0.005	<0.001
**Men-related journal comparisons**						
Favorability	0.394	0.216	0.049	–	–	–
Quality	0.164	0.053	0.145	–	–	–
Subscription Recommendations	0.170	0.024	0.134	–	–	–

#### Testing the Subscription Recommendations Explained by Androcentric Evaluative Bias Hypothesis?

Following Judd et al.’s (2001) mediational recommendations for within-participants designs, we examined whether the computed difference between sex/gender-related or men-related versus matched other-specialty psychology journals for subscription recommendations was predicted by the computed difference for favorability/quality ratings. Higher numbers favor the men or other-specialty journals. Participants’ decreased favorability/quality beliefs about sex/gender-related and men-related journals versus their matched other-specialty psychology journal(s) were associated with decreased subscription recommendations [Sex/gender-related, Study 1: favorability: *b* = 22.57, β = 0.86, *t*(108) = 17.66, *p* < 0.001 (95% CI 20.038 – 25.104), quality: *b* = 0.91, β = 0.80, *t*(106) = 13.54, *p* < 0.001 (95% CI 0.779 – 1.046), Study 2: favorability: *b* = 17.97, β = 0.81, *t*(422) = 27.95, *p* < 0.001 (95% CI 16.708 – 19.235), quality: *b* = 0.79, β = 0.79, *t*(421) = 26.28, *p* < 0.001 (95% CI 0.729 – 0.847); Men-related, Study 1: favorability: *b* = 25.07, β = 0.78, *t*(107) = 12.78, *p* < 0.001 (95% CI 21.177 – 28.954), quality: *b* = 0.90, β = 0.62, *t*(105) = 8.03, *p* < 0.001 (95% CI 0.676–1.119)].

##### Gender Effects

We examined whether these patterns emerged for the interactions between journal type and gender (0 = women, 1 = men) for sex/gender-related versus their matched other-specialty journals.^1^ Men’s decreased favorability/quality beliefs were associated with decreased subscription recommendations for sex/gender-related versus other-specialty psychology journals [Study 1: favorability: *b* = 23.67, β = 0.86, *t*(108) = 17.63, *p* < 0.001 (95% CI 21.006 – 26.327), quality: *b* = 0.99, β = 0.79, *t*(106) = 13.43, *p* < 0.001 (95% CI 0.842 – 1.134); Study 2: favorability: *b* = 18.50, β = 0.78, *t*(414) = 25.51, *p* < 0.001 (95% CI 17.075 – 19.926), quality: *b* = 0.83, β = 0.83, *t*(413) = 30.77, *p* < 0.001 (95% CI 0.782 – 0.888)].

##### Feminist Ideology Effects

We also examined whether these patterns emerged for feminist ideology. For people low in feminist ideology, decreased favorability/quality beliefs were associated with decreased subscription recommendations for sex/gender-related and men-related versus their matched other-specialty psychology journal(s) [Sex/gender-related, Study 1: favorability: *b* = 19.07, β = 0.78, *t*(107) = 12.86, *p* < 0.001 (95% CI 16.134 – 22.014), quality: *b* = 0.98, β = 0.80, *t*(105) = 13.67, *p* < 0.001 (95% CI 0.835 – 1.112), Study 2: favorability: *b* = 17.08, β = 0.78, *t*(419) = 25.75, *p* < 0.001 (15.777 – 18.385), quality: *b* = 0.80, β = 0.81, *t*(418) = 27.83, *p* < 0.001 (95% CI 0.745 – 0.858); Men-related, Study 1: favorability: *b* = 23.31, β = 0.81, *t*(107) = 14.45, *p* < 0.001 (95% CI 20.11 – 26.51), quality: *b* = 0.84, β = 0.69, *t*(105) = 9.76, *p* < 0.001 (95% CI 0.671 – 1.013)].

#### Summary of Findings

Despite a narrow content focus and equal impact ratings, undergraduate students enrolled in psychology classes demonstrated androcentric evaluations of sex/gender-related psychology journals. Partially consistent with the overall *androcentric bias hypothesis*, in Study 2 sex/gender-related psychology journals were judged as less meritorious (as Study 2 was the only sample with sufficient power to detect this effect). Further, the men-related journal was rated as less meritorious than its matched other-specialty journal but was perceived more favorably than the other sex/gender-related journals (no differences emerged for quality or subscription recommendations) suggesting a smaller penalty against men-related research outlets (Study 1 had sufficient power to detect effects for the men-related journal).

Most importantly, both samples had sufficient power to test the *gender differences in androcentric bias hypothesis*. Partially consistent with the gender differences in androcentric bias hypothesis, sex/gender-related psychology journals were judged by undergraduate men as less favorable, expressed lower subscription recommendations (Studies 1–2), and of lower quality (Study 2) than other-specialty journals. In Studies 1–2, undergraduate women perceived sex/gender-related journals more favorably than other-specialty journals but equally on quality and subscription recommendations. No gender differences emerged in the evaluation of the men-related psychology journal (in comparison to its other-specialty journal). Gender differences only emerged for favorability when comparing the men-related psychology journal to the sex/gender-related psychology journals with undergraduate men evaluating the sex/gender-related journals less favorably than the men-related psychology journal (no differences emerged for undergraduate women).

Not only were women more likely than men to endorse feminist ideology, but, consistent with the *personal ideology differences in androcentric bias hypothesis*, the other-specialty journals had a weaker correlation with feminist ideology than the sex/gender-related journals (and the men-related journal for favorability only). Thus, individuals who were high in feminist ideology were also more likely to perceive the sex/gender-related journal as more favorable, of higher quality, and were more likely to recommend subscription maintenance.

Importantly, consistent with the *subscription recommendation explained by androcentric evaluative bias hypothesis*, decreased favorability/quality beliefs about sex/gender-related or men-related journals versus their other-specialty journals predicted decreased library subscription recommendations. This pattern was especially pronounced for men (for sex/gender-related journals only) and people low in feminist ideology (Studies 1–2).

Despite critical limitations in Study 1 [e.g., underpowered to detect the main effect of journal for the sex/gender-related journal comparisons, the journals were chosen based on the 1-year impact factor, and the matched journals confounded gender with class (*Military Psychology*) and race (*Journal of Psychology in Africa*)], our results were generally replicated in Study 2. In Study 2, we tripled our participant population, chose journals based on their 5-year impact factor (a less variable measure of journal quality/prestige), and controlled for race and class in our selection of other-specialty journals.

Results suggest the existence of at least some androcentric biases among undergraduate men in psychology. What might people outside the field of psychology perceive? On the one hand, the overall androcentric bias hypothesis would predict the same expressions of bias no matter the audience; sex/gender-related journals would be devalued. However, perhaps people outside of psychology, who do not experience the gendered power-difference within psychology, only see a field that is now dominated by women, and therefore do not distinguish the different types of psychology journals from one another but instead assume all psychology-related topics are “feminine.” On the other hand, it is also possible that public engagement with sex/gender-related journal articles would be greater than other-specialty journal articles as a form of androcentric interest; anything confirming or challenging androcentrism might be more likely to capture public attention. We examined these hypotheses in Study 3, by analyzing popular press metrics using Altmetrics. Altmetrics is a unique index of research impact (Kwock, 2013) because of the ever-growing role that social media plays in research dissemination (Sugimoto et al., 2017). While research article visibility on social media has a small positive correlation with citation count, visibility and citation count are distinct metrics (Costas et al., 2015). However, the same gender biases that emerge in traditional article metrics (like citation count; Larivière et al., 2013) also emerge for online visibility. For instance, male-identified scientists received more attention than female-identified scientists among the top 25% of online scholars, regardless of the research area and the proportion of female-identified scientists in the research area (Vasarhelyi et al., 2021). Thus, androcentric biases occur overall in the dissemination of online scholarship because people pay more/less attention to research based on the authors’ characteristics (gender, race, university affiliation, e.g., Vasarhelyi et al., 2021). But what we do not know is will research within psychology about sex/gender similarly be ignored or, perhaps, receive extra interest because it confirms or challenges the *status quo*. With this ambiguity in mind, Study 3 documented the public reach of articles published in sex/gender-related versus matched other-specialty psychology journals.

## Study 3

### Articles Selected From Journals

The top 50 articles published between the date the journal was created and July 2021 were selected from all of the sex/gender-related psychology journals and the other-specialized journals used in Studies 1–2.

### Article Reach

Altmetrics examines the social impact of a journal through mentions of the journal in the popular press at the level of the article (Wee and Chia, 2014) and includes “peer reviews on Faculty of 1000, citations on Wikipedia and in public policy documents, discussions on research blogs, mainstream media coverage, bookmarks on reference managers like Mendeley, and mentions on social networks such as Twitter” (Altmetric, 2018). A higher Altmetrics score (any number between 0 to ∞) suggests an article has more public reach. Altmetrics excludes shares when the original research is not linked or inconsistent hashtags are used (Taylor, 2013). Though particular sources (e.g., tweets, blogs, etc.) can be analyzed separately, the inflation of alpha and the sheer number of sources, warranted the examination of only the overall Altmetrics score.

## Results

First, we analyzed the Altmetrics score of the top 50 articles from the four matched journals grouped by Study 1 and Study 2 by submitting the articles to one-way between-articles ANOVAs. Next, we separately analyzed the top 50 articles from each of the four sex/gender-related journals in comparison with their respective matched journal from Studies 1 and 2 by submitting articles to one-way between-articles ANOVAs contrasting each sex/gender-related journal with each of its two comparable journals (Table 9).

**TABLE 9:** ANOVAs comparing articles published in sex/gender-related versus other matched specialty journals: Study 3.

Overall Comparisons																				

	Journal Comparisons from Study 1 (*df*: 1, 398)					Journal Comparisons from Study 2 (*df*: 1, 398)														
	*F*	*p*	*d*	*M*	*SD*	*F*	*p*	*d*	*M*	*SD*										
Journal type	1.19	0.276	0.109	–	–	95.59	<0.001	−0.978	–	–										
Sex/gender-related	–	–	–	134.67	154.43	–	–	–	134.67_*a*_	154.43										
Other-specialty	–	–	–	161.68	313.86	–	–	–	18.77_*b*_	65.26										

**Comparisons by Journal**																				

	***Women and Therapy* Comparisons (*df*: 2, 147)**					***Feminism and Psychology* comparisons (*df*: 2, 147)**					***Sex Roles* Comparisons (*df*: 2, 147)**					***Psychology of Women Quarterly (*df*: 2, 147)* Comparisons**				
				
	***F***	***p***	***ds***	***M***	***SD***	***F***	***p***	***ds***	***M***	***SD***	***F***	***p***	***ds***	***M***	***SD***	***F***	***p***	***ds***	***M***	***SD***

Journal Type	12.58	<0.001	−0.523 to 0.482	–	–	33.18	<0.001	−1.326 to 0.690	–	–	32.92	<0.001	−1.541 to 0.113	–	–	50.35	<0.001	−2.300 to 1.610	–	–
Sex/gender-related	–	–	–	20.10_*a*_	24.57	–	–	–	63.32_*a*_	62.51	–	–	–	303.28_*a*_	195.34	–	–	–	151.98_*a*_	79.28
Study 1 other-specialty	–	–	–	8.82_*b*_	18.11	–	–	–	15.52_*b*_	22.06	–	–	–	104.50_*b*_	163.81	–	–	–	517.88_*b*_	440.41
Study 2 other-specialty	–	–	–	2.44_*b*_	4.67	–	–	–	4.64_*b*_	3.24	–	–	–	52.44_*b*_	121.92	–	–	–	15.54_*c*_	27.49

*Different subscripts within a dependent measure differ from each other, p < 0.05. The convention of Cohen’s d was used: <0.20 a small effect, 0.20 to 0.80 a moderate effect, >0.80 a large effect. Effect sizes with positive numbers indicate differences favoring other-specialty journals.*

### Study Level Article Reach Journal Comparisons

Although for the Study 1 comparisons no differences on Altmetrics score emerged between the top 50 articles from sex/gender-related psychology journals and other-specialty psychology journals, *F*(1,398) = 1.09, *p* = 0.275, *d* = 0.109 (95% CI –75.636 – 21.616), for the Study 2 comparisons, the top 50 articles in sex/gender-related journals were more likely to have higher Altmetrics scores than their matched top 50 articles in other-specialty journals, *F*(1,398) = 95.59, *p* < 0.001, *d* = –0.978 (95% CI 92.599 – 139.211).

### Comparisons Between the Sex/Gender Journal and Its Matched Journals

Given the inconsistent results when other-specialty journals were collapsed into a single category, we conducted follow-up analyses comparing each sex/gender-related journal to each corresponding other-specialty journal comparison.

#### Women and Therapy Comparisons

Contrasting the article results from *Women and Therapy* compared with *Journal of Psychology in Africa* (matched 1-year impact factor) and *Psychologia* (matched 5-year impact factors), a main effect emerged for Altmetrics score [*F*(2,147) = 12.58, *p* < 0.001]. Articles in *Women and Therapy* received higher Altmetrics scores than articles in the *Journal of Psychology in Africa* [*p* = 0.002, *d* = –0.523 (95% CI 4.233 – 18.327)] and *Psychologia* [*p* < 0.001, *d* = –0.999 (95% CI 10.613 – 24.707)]. Articles in the *Journal of Psychology in Africa* and *Psychologia* did not differ on Altmetrics score [*p* = 0.076, *d* = 0.482 (95% CI –0.667 – 13.427)].

### Feminism and Psychology Comparisons

Contrasting the article results from *Feminism and Psychology* compared with *Military Psychology* (matched 1-year impact factor) and *Journal of Classification* (matched 5-year impact factors), a main effect emerged for Altmetrics score [*F*(2,147) = 33.18, *p* < 0.001]. Articles in *Feminism and Psychology* received higher Altmetrics scores than articles in the *Journal of Classification* [*p* < 0.001, *d* = –1.326 (95% CI –73.825 to –43.535)] and *Military Psychology* [*p* < 0.001, *d* = –1.020 (95% CI 32.655 – 62.945)]. Articles in the *Journal of Classification* and *Military Psychology* did not differ on Altmetrics score [*p* = 0.158, *d* = 0.690 (95% CI –26.025 – 4.265)].

#### Sex Roles Comparisons

Contrasting the article results from *Sex Roles* compared with *Group Processes and Intergroup Relations* (matched 1-year impact factor) and *Thinking and Reasoning* (matched 5-year impact factors), a main effect emerged for Altmetrics score [*F*(2,147) = 32.92, *p* < 0.001]. Articles in *Sex Roles* received a higher Altmetrics score than articles in *Group Processes and Intergroup Relations* [*p* < 0.001, *d* = –1.103 (95% CI 134.294 – 263.266)] and *Thinking and Reasoning* [*p* < 0.001, *d* = –1.541 (95% CI 186.354 – 315.326)]. *Group Processes and Intergroup Relations* did not differ from *Thinking and Reasoning* on Altmetrics score [*p* = 0.113, *d* = 0.361 (95% CI –12.426 – 116.546)].

#### Psychology of Women Quarterly Comparisons

Contrasting the article results from *Psychology of Women Quarterly* compared with *Personality and Individual Differences* (matched 1-year impact factor) and *European Journal of Psychological Assessment* (matched 5-year impact factors), a main effect emerged for Altmetrics score [*F*(2,147) = 50.35, *p* < 0.001]. Articles in *Psychology of Women Quarterly* received a higher Altmetrics score than articles in the *European Journal of Psychological Assessment* [*p* = 0.009, *d* = –2.300 (95% CI –238.748 to –34.132)]. Articles in *Personality and Individual Differences* received a higher Altmetrics score than articles in the *European Journal of Psychological Assessment* [*p* < 0.001, *d* = 1.610 (95% CI –604.64 to –400.032)]. Interestingly, *Psychology of Women Quarterly* received a lower Altmetrics score than articles in *Personality and Individual Differences* [*p* < 0.001, *d* = 1.156 (95% CI –468.208 to –263.592)].

### Discussion

Findings from Study 3 illustrate that, despite being perceived as lower quality by undergraduate men within psychology (Studies 1–2), articles in sex/gender-related psychology journals have, on average, greater public reach through shares in social media and the popular press. For the most part, articles from sex/gender-related journals were more likely to have higher Altmetrics scores than their matched other-specialty journals with one exception: articles published in *Personality and Individual Differences* did have a higher Altmetrics score when compared with articles published in the *Psychology of Women Quarterly.* These results suggest a possible novel conceptualization of what might be called *androcentric-interest*; greater attention to sources or topics that may confirm or challenge androcentrism. It is difficult to know why an article is shared (or cited for that matter); it could be for example that articles about sex/gender might provoke extra scrutiny because of findings that are challenging the (androcentric) *status quo* and that extra scrutiny takes the shape of public sharing. Or it could be that an article is shared because the findings are exciting or unexpected – even when the finding fails to replicate (O’Grady, 2021). In both cases, an androcentric-interest proclivity could be in play, but for very different reasons. Of course, this is a very preliminary interpretation of the current results and much more future research is needed to flush out this concept.

## General Discussion

Our research advances knowledge about androcentrism (e.g., Bailey et al., 2019) by examining whether androcentric bias emerges in perceptions of research outlets within a field that studies androcentrism. The result is a complicated picture that depends on the audience (if the person is in close contact with the field versus the general public) and the method of evaluation.

The androcentric bias against sex/gender-related psychological research was clearer when evaluators were enrolled in psychology coursework. When examining judgments by undergraduate students, sex/gender-related (and, to some degree, men-related) journals were viewed as less meritorious. This was mostly driven by the biases held by undergraduate men (for sex/gender-related journals) and people low in feminist ideology (Studies 1–2). Undergraduate women and people high in feminist ideology were generally more favorable toward sex/gender-related psychology journals (Studies 1–2). Interestingly, undergraduate students also judged the men-related journal as less meritorious than its matched other-specialty journal but more favorably than the sex/gender-related journals (Study 1). Favorability and quality judgments accounted for the low subscription recommendations for the sex/gender-related journals and the men-related journal, especially among men (for the sex/gender-related journals) and people low in feminist ideology (Studies 1–2). Importantly, androcentric bias emerged regardless of whether participants read the actual or modified descriptions from the journals’ websites.

However, when looking at a very different form of engagement with sex/gender journals within psychology, Altmetrics, results showed that the top 50 articles published in sex/gender-related journals received more public attention (sharing, news reports) on average than their matched other-specialty journals (Study 3). Though these data are only descriptive, the results set up a fruitful line of future work to understand why people share certain articles over others. We speculate that perhaps something akin to an androcentric-interest proclivity is operating such that people are especially attuned to research about sex/gender because the results may either support or refute the very nature of androcentric tendencies.

### Implications

We know that when a person is in an environment where they are frequently exposed to more men in power, such as academia, androcentric bias is especially likely to emerge (Bailey et al., 2019). Though psychology as a field is more women-dominated over time (National Science Foundation [NSF], 1993, 2015), the levers of men’s privilege and power are still evident (Klatzky et al., 2015; Vaid and Geraci, 2016). As a field that studies androcentrism, stereotypes, and prejudice, it is ironic that people learning about psychology would reproduce the very bias it studies. Though we only studied students engaging in psychology coursework, the implication for the faculty teaching and mentoring those students, working in those universities, and indeed the next generation of scholars is worrisome. For example, likely, these patterns would also emerge in populations with greater academic training in psychology (graduate students, postdoctoral researchers, and junior faculty; Nylenna et al., 1994; Kliewer et al., 2005; Borsuk et al., 2009). The presence of the bias within the very field that studies it, also speaks to the importance of integrating content and training related to diversity, equity, and inclusion (including intersectional feminism) within the curriculum even when that curriculum on the surface should already include such topics. Indeed, previous research demonstrates that exposure to feminism results in increased feminist identification (Henderson-King and Stewart, 1999; Reid and Purcell, 2004). As undergraduate psychology students are the future of psychology, our study provides an initial critical exposure point and identifies another important form of androcentric bias; research outlet favoritism.

We anticipate that androcentric bias against psychology journals specializing in sex/gender research is problematic for students who complete work related to the psychology of women and gender studies. Not only might women and gender studies degrees be perceived as less valuable, an important question for future research, but the relative dismissal or neglect of the psychology of sex/gender research is disconcerting to the extent that such knowledge is informative and useful to advancing discovery, innovation, and creativity in other disciplines. The exclusion of one type of knowledge, especially by those higher in social standing, such as men, feeds into the *status quo* of what “counts” as knowledge (e.g., Harding, 1991).

This androcentric bias might even be problematic at the faculty level. Since hiring and tenure decisions are, in part, based on the (perceived) prestige of a candidate’s publications (Steinpreis et al., 1999), our findings potentially paint a troubling picture for social scientists who study sex/gender, who are disproportionately women (American Psychological Association [APA], 2006). Men, albeit undergraduate students in our study, were especially likely to disparage sex/gender-related journals but women more favorably evaluated sex/gender-related journals. Might these preferences cancel out men’s disparaging tendencies? The answer is likely no, as men are overrepresented as tenure-track and tenured psychology professors in the United States (Oklahoma State University [OSU], 2011). Some of the undergraduates evaluating these journals will 1 day be in tenure-track and tenured positions. If men reviewing job applications and tenure dossiers are unaware of their androcentric biases, we anticipate they might undervalue research published in sex/gender-related journals. The cumulative negative downstream implications of undervaluing these journals could include employment, graduate school enrollment, retention in faculty positions, promotion, awards, raises, other resources, and accolades for students and psychological scholars of sex and gender.

However, our findings do point to a way to decrease this androcentric bias. By including Altmetrics data (or other data about public reach) within evaluation materials and giving this public reach data equal weight, topics published in sex/gender-related journals will be evaluated as more meritorious since articles published in sex/gender-related journals received on average more public reach than articles published in matched other-specialty journals. Our findings also highlight that androcentric-interest might occur within the general public which points to an avenue of potentially educating and exposing the public to this type of androcentric bias research to help decrease androcentrism more broadly.

### Limitations and Future Directions

The current research examined two undergraduate student samples’ evaluations of sex/gender-related (and men-related, Study 1) journals and the Altmetrics of articles published in sex/gender-related journals. Although the current studies found that subscription recommendations were accounted for by low favorability and quality perceptions, especially for men (for sex/gender-related journals) and individuals low on feminist ideology, it is unclear what is driving these low favorability and quality ratings. Future research should address whether these decreased favorability and quality perceptions occurred because it is assumed that the researchers are women, the participants are women, or because the findings are assumed to be pro-woman or feminist. If the researchers are assumed to be women they might be subject to stereotypes that researchers are engaging in “me-search” (Rios and Roth, 2020) or that women are scientifically less competent (Moss-Racusin et al., 2012). If participants are assumed to be women or if the findings are assumed to be pro-woman/feminist the findings might be subject to the belief that the results are not broadly generalizable (which would be further evidence of androcentric bias). Only future research will help answer these important questions.

Further, we predict the marginalization of knowledge is especially pronounced when people are within environments where men are overrepresented within positions of power (Bailey et al., 2019) and within personally meaningful situations (Petty and Cacioppo, 1986). Future research could also examine when knowledge related to sex and gender is most likely to be marginalized among people within other domains that are also men-dominated, at different career stages within an academic domain (undergraduate, graduate, postdoctoral, junior faculty, and senior faculty), and at different stages of knowledge about feminism. Does knowledge about the field of study, previous exposure to sex/gender-related journals, as well as education about feminism moderate the effect?

In Studies 1–2 we found that the correlation between the ratings of the other-specialty journals and feminist ideology was weaker than the correlation between the ratings of sex/gender-related journals (and for the men-related journal’s favorability ratings) and feminist ideology. However, a critical limitation of this finding was that we also found that women were more likely to endorse feminist ideology than men. Previous research suggests that ideological differences such as egalitarianism (Plant and Devine, 1998; Crandall et al., 2002) and sexist ideology (i.e., Swim et al., 2004; Sczesny et al., 2015) moderate findings of androcentrism. However, it is unclear from our finding whether men are less favorable toward sex/gender-related journals because they scored lower in feminist ideology. We suspect that this is not the case as past research on androcentrism does not consistently show participant gender effects (Harding, 1991) and in our sample there was only small correlation between gender and feminist ideology [Study 1: *r*(109) = –0.22, *p* = 0.020; Study 2: *r*(417) = –0.26, *p* < 0.001]. Future research would do well to further unpack these findings by examining the relationship between gender, feminist ideology, and androcentric bias.

It is important to point out that while Studies 2 and 3 controlled for race and class within the journal titles, we did not control for nationalism. Studies 1 and 3 included the *Journal of Psychology in Africa* while Studies 2 and 3 included the *European Journal of Psychological Assessment*. The word “Africa” might evoke racial stereotypes and nationalism whereas the word “European” might evoke nationalism. Because most psychological research is done in Western societies (Henrich et al., 2010), perhaps among U.S. participants, journals with the term “European” evoked similar nationalism to journals with no geographical references among our U.S. study samples.

It is also possible that because the undergraduate students in Studies 1 and 2 were not experts in psychology, they perceived the other-specialty journals as being more generalized than the more obviously “specialized” sex/gender-related journals. While we did not ask the undergraduate students about how specialized they perceived the journals to be, differential perceptions of specialization may be part of the marginalization process. Future research could examine under what conditions journals and knowledge related to sex and gender is more likely to be considered equally or more “specialized” than other types of journals and knowledge.

It is true that we only examined responses to and the public reach of sex/gender-related journals in psychology and did not examine whether sex/gender-related articles published in non-gender journals or non-psychology-related journals suffer similar fates. We suspect that they might as when scientists, especially men, read an abstract about gender bias within science, they perceived the research less favorably and of lower quality (Handley et al., 2015). When journals specialize in diversity research and when research on similar topics is published, key findings on social change likely have reduced impact within a field but could have more impact within the public. Future research should examine whether research on gender published in non-gender journals and/or in non-psychology-related journals is perceived as less meritorious in an area of study but as of more interest within the general public.

There are many explanations for why the marginalization of knowledge related to sex and gender exists primarily among undergraduate men and people low in feminist ideology. Future research should uncover whether hindsight bias (Hawkins and Hastie, 1990), lay-theories about feminine fields requiring less innate talent (Leslie et al., 2015), and/or lower quality evaluations of research that does not support scientists’ prior beliefs (Koehler, 1993) underlie these processes. Future research should consider ways of making knowledge related to sex and gender more highly respected within psychology by examining the role of introductory psychology curriculum as well as diversity, equity, and inclusion curriculum requirements in this bias and comparing other sex/gender-related publications in other disciplines, such as sociology, history, and political science, with those of psychology.

In Study 1, we found that sex/gender-related journals but also, in some cases, the men-related journal was viewed by undergraduate students as less meritorious than their matched other-specialty journals. These differential journal evaluations were particularly pronounced for people low in feminist ideology, which supports the idea that if a journal focuses on any aspect of sex or gender, it is marginalized to some degree. Consistent with the movement to think about gender issues in a less binary matter (Croft et al., 2015), future research should more robustly examine how men-related journals fare against matched other-specialty journals and other sex/gender-related journals to examine whether these journals are believed to be more, less, or equivalently meritorious in comparison with sex/gender-related journals as well as what topics within sex/gender-related and men-related journals are especially marginalized. Moreover, there is likely compounded bias within sex/gender research scholarship that focuses on intersectionality, where different vectors of power and access are analyzed as a function of the lived experiences of people with multiple identities (e.g., Hill Collins and Bilge, 2020).

## Conclusion

As psychological scientists, we are experts at studying, teaching, and sometimes translating to the public the complexities of explicit and subtle bias. College men and people low in feminist ideology marginalized these sex/gender-related psychology journals. Yet, the public was also much more interested in sharing research from these very journals. Such findings give name and perspective to a possibly emerging problem that serves as a call to action for publishers, students, faculty, and change agents who we hope will realize this androcentric bias exists and actively work to overcome it, including rethinking citation counts as an index for quality. Does the androcentric bias subside over time? Or does it intensify only for those for whom the topic is most relevant (Handley et al., 2015)? Why does the public have more interest in sharing sex/gender research and yet it is denounced among the people studying within the field itself? The current project lays the groundwork for more research on perceptions of sex/gender-related knowledge production and dissemination that impact the full participation and appreciation of scholars within the social sciences.

## Data Availability Statement

The raw data supporting the conclusions of this article will be made available by the authors, without undue reservation.

## Ethics Statement

The studies involving human participants were reviewed and approved by Institutional Review Board at Montana State University and the Institutional Review Board at the University of North Florida. The participants provided their written informed consent to participate in this study.

## Author Contributions

EB took the lead on all tasks related to the manuscript. EB and JS were involved with the study design, data collection, data analysis, and drafting of the manuscript. DR was involved in the data collection of the Altmetrics data and editing the document. All the authors contributed to the article and approved the submitted version.

## Conflict of Interest

The authors declare that the research was conducted in the absence of any commercial or financial relationships that could be construed as a potential conflict of interest.

## Publisher’s Note

All claims expressed in this article are solely those of the authors and do not necessarily represent those of their affiliated organizations, or those of the publisher, the editors and the reviewers. Any product that may be evaluated in this article, or claim that may be made by its manufacturer, is not guaranteed or endorsed by the publisher.

## Footnotes

1 Because no significant Participant Gender × Journal Type interaction emerged for the men-related versus matched other-specialty journal comparisons, we did not test whether gender moderated the mediation for men-related journal comparisons.
